# Detection of Extended-Spectrum β-Lactamases (ESBLs) and AmpC in Class A and Class B Carbapenemase-Producing *Enterobacterales*

**DOI:** 10.1128/spectrum.02137-22

**Published:** 2022-10-26

**Authors:** Frank Imkamp, Natalia Kolesnik-Goldmann, Elias Bodendoerfer, Reinhard Zbinden, Stefano Mancini

**Affiliations:** a Institute of Medical Microbiology, University Zurich, Zurich, Switzerland; Louis Stokes Cleveland VAMC

**Keywords:** AmpC detection, carbapenemase-producing *Enterobacterales*, disc diffusion, ESBL detection

## Abstract

In carbapenemase-producing *Enterobacterales* (CPE) additional β-lactam resistance mechanisms such as extended-spectrum-β-lactamases (ESBL) and/or AmpC-β-lactamases are generally difficult to detect by phenotypical methods. Recently, a modified version of the CLSI ESBL confirmatory combination disc diffusion (CDD) test, which involves the addition of boronic acid and EDTA on discs containing ESBL and AmpC substrates ± inhibitors, has been proposed for the detection of ESBL in class A and class B CPE. Here, the performance of the modified CDD test was evaluated using 121 genotypically characterized class A and class B CPE. Also, the effectiveness of the NG-Test CTX-M-MULTI lateral flow immunoassay was evaluated for ESBL detection. For class A CPE (*n* = 47), the modified CDD method exhibited an equal specificity (95.7%) and a higher sensitivity (100%) compared to the standard method (91.7%). The CTX-M-MULTI test detected ESBL in all CTX-M-type ESBL producers (*n* = 23), whereas it was negative for all CTX-M-type ESBL-negative isolates (*n* = 24). For class B CPE (*n* = 71), the modified method significantly improved both sensitivity (95%) and specificity (100%) in detecting ESBL compared to the standard method (17.5% sensitivity and 83.9% specificity). In comparison, the CTX-M-MULTI led to identification of ESBL in all CTX-M-ESBL-producers (*n* = 39) and no false-positive signal was generated with the CTX-M-type-ESBL-negative isolates (*n* = 30). Furthermore, the modified CDD improved the robustness of the method for AmpC detection (inconclusive results were produced in 53/57 and 10/57 cases with the standard and modified method, respectively), although the sensitivity of the test was poor (23.5%). Here, we propose a practical and cost-effective approach combining the modified CDD and the CTX-M-MULTI test for detection of ESBL and/or AmpC in class A and B CPE.

**IMPORTANCE** Antimicrobial resistance is a growing public health threat of broad concern worldwide. Timely detection of antibiotic resistance mechanisms can help to monitor and to curb the spread of resistant bacteria within the hospital setting as well as in the environment. In this work we report an accurate and affordable method to phenotypically identify difficult-to-detect resistance determinants in highly resistant (carbapenemase-producing) bacteria. This method may be implemented in any diagnostic microbiology lab and may reduce the underreporting of relevant resistance mechanisms.

## INTRODUCTION

The spread of multidrug-resistant *Enterobacterales* decreases the available options for active empirical treatment of infections caused by these organisms. Bacterial strains producing extended-spectrum-β-lactamases (ESBL) and AmpC β-lactamases are of particular concern, as they are resistant to classical first-line antibiotics. ESBLs are mostly plasmid-mediated β-lactamases that confer resistance toward aminopenicillins, oxyimino-cephalosporins (e.g., ceftriaxone, ceftazidime, cefotaxime, and cefepime), and monobactams. Yet, ESBLs are inhibited by β-lactamase inhibitors such as clavulanic acid and tazobactam (1). AmpC β-lactamases can efficiently hydrolyze aminopenicillins, oxyimino-cephalosporins (e.g., ceftriaxone, ceftazidime, cefotaxime, but not cefepime), cephamycins (e.g., cefoxitin), and monobactam, while they are resistant to ESBL inhibitors (2). Production of AmpC in *Enterobacterales* is mediated either by chromosomal determinants or resistance plasmids. Some *Enterobacterales* species, such as Enterobacter cloacae, Klebsiella aerogenes, Citrobacter freundii, or Serratia marcescens, carry a chromosomal *ampC* gene, which is normally expressed at low levels but can be induced by β-lactams such as cefoxitin and imipenem. Also, mutations in the regulatory gene *ampD* or in the promoter/attenuator region of *ampC* in Escherichia coli may lead to constitutive overproduction of AmpC. Today, there are many diagnostic tools, including phenotypic and genotypic tests that allow prompt and accurate detection of ESBL and AmpC β-lactamases (3). Detection of these resistance mechanisms constitutes an important part of the epidemiological monitoring and surveillance of antimicrobial resistance.

The European Committee on Antimicrobial Susceptibility Testing (EUCAST) recommends different phenotypic or biochemical tests to confirm the presence of suspected ESBL and/or AmpC β-lactamases (4). A widely used phenotypic confirmatory method is the combination disc diffusion (CDD) test, which is based on the increase of inhibition zone diameters caused by ESBL or AmpC inhibitors (clavulanic acid or cloxacillins, respectively) around discs containing cephalosporins (ceftazidime, cefotaxime, and cefepime) or cephamycins (cefoxitin or cefotetan). However, this test can be affected by the simultaneous presence of other β-lactamases, such as class A (mostly of Klebsiella pneumoniae carbapenemase [KPC]-type) and class B (mostly metallo-β-lactamases [MBL] of type NDM, VIM, and IMP) carbapenemases, which can also hydrolyze extended-spectrum β-lactams without being affected by ESBL- and/or AmpC-inhibitors. Conversely, for class D carbapenemases, which can only weakly hydrolyze cephalosporins, the CDD test can adequately detect ESBLs (5).

Modified methods based on the addition of class A and class B carbapenemase inhibitors (boronic acid and EDTA, respectively) on the antibiotic discs containing cephalosporins or cephamycins with and without the ESBL and/or AmpC inhibitors have been reported in the literature (5, 6). For ESBL detection, other methods, including biochemical tests based on the detection of cefotaxime hydrolysis that is inhibited by tazobactam, or lateral flow immunoassays (LFIA) based on antibody-mediated detection of ESBL antigens, have been recently reported (7, 8). For instance, the NG-Test CTX-M-MULTI (hereafter CTX-M-MULTI) is a rapid, easy-to-perform, and cost-effective LFIA allowing the detection of group 1, 2, 8, 9, and 25 CTX-M producers from pure cultures grown on agar plates.

In this study, we aimed to evaluate the diagnostic performances of the modified ESBL and AmpC confirmatory CDD tests for the detection of ESBL in class A carbapenemase-producing *Enterobacterales* (CPE) and ESBL and AmpC in class B CPE. Moreover, we evaluated the performance of the CTX-M-MULTI test and propose a practical approach for confirmation of ESBL and/or AmpC production in class A and class B CPE in a clinical microbiology laboratory.

## RESULTS

### Genotypic analysis.

The strain collection used in this study included 47 KPC-, 71 class B MBL-, and 3 KPC/MBL-producing CPE (Table 1). Genotypic analysis revealed that among the KPC-producing CPE, 24 were positive for ESBL. Of the 24 ESBLs, 19 were CTX-M-15, 1 was CTX-M-55, and 1 was SHV-66. One isolate contained both a CTX-M-15 and a CTX-M-65 ESBL, another strain harbored both a CTX-M-55 and a SHV-66 ESBL, and another one carried a CTX-M-15 and an SHV-66 ESBL in addition to a plasmidic CMY-16 AmpC. Of the 23 ESBL nonproducers, 3 were intrinsic AmpC-producers (C. freundii) and plasmidic AmpC β-lactamases were not detected in any of the 23 isolates.

**TABLE 1:** Distribution of ESBL and AmpC genes among the carbapenemase-producing *Enterobacterales* used in this study

Strain	K. pneumoniae	E. coli	E. cloacae	C. freundii	P. mirabilis	P. stuartii	P. rettgeri	S. marcescens	K. oxytoca	Total
KPC producers (*n* = 47)	40	4		3						47
ESBL producers (*n* = 24)	23	1								24
ESBL non-AmpC-producers (*n* = 23)	22	1								23
*bla*_CTX-M-15_	19									19
*bla*_SHV-66_	1									1
*bla*_CTX-M-55_	1									1
*bla*_CTX-M-55_ + *bla*_SHV-66_	1									1
*bla*_CTX-M-15_ + *bla*_CTX-M-65_		1								1
ESBL + plasmidic AmpC producers (*n* = 1)	1									1
*bla*_CYM-16_ + *bla*_CTX-M-15_ + *bla*_SHV-66_	1									1
Non-ESBL producers (*n* = 23)	17	3		3						23
Non-ESBL and non-AmpC producers (*n* = 20)	17	3								20
Non-ESBL intrinsic AmpC-(hyper) producers (*n* = 3)				3						3
Total	40	4	0	3	0	0	0	0	0	47
MBL producers (*n* = 71)	34	20	8	3	1	2	0	1	2	71
ESBL producers (*n* = 40)	24	6	6		1	2			1	40
ESBL and non-AmpC producers (*n* = 21)	14	5			1				1	21
*bla*_CTX-M-15_	14	4								18
*bla*_CTX-M-24_		1								1
*bla*_CTX-M-9_									1	1
*bla*_CTX-M-2_ + *bla*_CTX-M-8_					1					1
ESBL and plasmidic AmpC producers (*n* = 11)										
*bla*_CYM-2_ + *bla*_CTX-M-15_		1								1
*bla*_CYM-4_ + *bla*_CTX-M-15_	1									1
*bla*_CYM-6_ + *bla*_CTX-M-15_	3									3
*bla*_CYM-16_ + *bla*_CTX-M-15_	4									4
*bla*_DHA-1_ + *bla*_CTX-M-15_	2									2
ESBL + intrinsic AmpC (hyper) producers (*n* = 8)			6			2				8
Intrinsic AmpC-producers + *bla*_CTX-M-2_ + *bla*_CTX-M-8_						2				2
Intrinsic AmpC-producers + *bla*_CTX-M-9_ + *bla*_SHV-12_			4							4
Intrinsic AmpC-producers + *bla*_CTX-M-15_			2					-		2
Non-ESBL producers (*n* = 31)	10	14	2	3				1	1	31
Non-ESBL and non-AmpC producers (*n* = 19)	8	10							1	19
Non-ESBL and plasmidic AmpC producers (*n* = 6)	2	4								6
*bla*_CYM-6_		1								1
*bla*_CYM-13_	1									1
*bla*_CYM-42_		3								3
*bla_DHA-1_*	1									1
Non-ESBL intrinsic AmpC (hyper) producers (*n* = 6)			2	3				1		6
Total	34	20	8	3	1	2	0	1	2	71
KPC and MBL producers (*n* = 3)	2						1			3
ESBL producers (*n* = 1)							1			1
ESBL + intrinsic AmpC (hyper) producers (*n* = 1)							1			1
Intrinsic AmpC producers + *bla*_PER-4_							1			
Non-ESBL producers (*n* = 2)	2									2
Non-ESBL and non-AmpC producers (*n* = 2)	2									
Total	2	0	0	0	0	0	1	0	0	3
Total overall	76	24	8	6	1	2	1	1	2	121

ESBLs were identified in 40 out of 71 MBL-producing CPE and all were CTX-M type (31 isolates with CTX-M-15, 1 with CTX-M-24, 1 with CTX-M-9, 3 with CTX-M-2/CTX-M-8, and 4 with CTX-M-9/SHV-12). Of the ESBL-positive MBL producers, 11 carried a plasmidic AmpC β-lactamase (9 of CMY type and 2 of DHA type), while 8 produced an intrinsic AmpC (6 E. cloacae and 2 Providencia stuartii isolates). Of the 31 ESBL nonproducers, 6 carried an intrinsic AmpC (1 S. marcescens, 2 E. cloacae and 3 C. freundii isolates) and 6 carried a plasmidic AmpC (5 of CMY type and 1 of DHA type).

Of the 3 CPE producing both a KPC and an MBL, 2 were neither ESBL producers nor AmpC producers, while the third isolate was an intrinsic AmpC producer (Providencia rettgeri) and was positive for a PER-4-ESBL.

### Standard EUCAST CDD results on KPC-producing CPE.

The standard EUCAST CDD test based on the diagnostic flow chart elaborated by Polsfuss et al. (9) allowed the detection of ESBLs in 22/24 (91.7%) ESBL-positive isolates (genotypically confirmed) belonging to the group of KPC-producing CPE (*n* = 47). Within the same group 22/23 (95.7%) ESBL-negative isolates were correctly classified as such (Tables 2 and 3). Of note, the two ESBL-positive isolates where ESBL was not detected with the standard CDD test harbored both a CTX-M-55 β-lactamase (in one case in combination with a SHV-66 ESBL). Furthermore, the only plasmidic AmpC (CMY-16) genotypically identified within the 45 KPC-producing CPE was not phenotypically detected. All 43 AmpC-negative KPC-producing isolates were correctly classified.

**TABLE 2:** Phenotypic detection of ESBL and AmpC among carbapenemase-producing *Enterobacterales* clinical isolates using the standard and modified confirmatory ESBL/AmpC tests^*a*^

Phenotypic confirmatory test			ESBL detection								AmpC detection							
			Standard				Modified				Standard				Modified			
Genotype			No. (%)	Sensitivity %	Specificity %	Inconclusive (%)	No. (%)	Sensitivity %	Specificity %	Inconclusive (%)	No. (%)	Sensitivity %	Specificity %	Inconclusive (%)	No. (%)	Sensitivity %	Specificity %	Inconclusive (%)
Carbapenemase(s)	ESBL/AmpC	No.																
KPC (*n* = 45)	ESBL pos/AmpC neg	22	21 (95.5%)	95.5			22 (100%)	100			0 (0)		100	10/22 (45.5)				
	ESBL pos/pAmpC pos	1	1 (100%)	100			1 (100%)	100			0 (0)	0		1/1 (100)				
	ESBL neg/AmpC neg	19	1 (5.3)		94.7		1 (5.3)		94.7		0 (0)		100	4/19 (21)				
	ESBL neg/gAmpC pos	3	0 (0)		100		0 (0)		100									
KPC/OXA-48-type (*n* = 2)	ESBL pos/AmpC neg	1	0 (0)	0			1 (100)	100			0 (0)		100	1/1 (100)				
	ESBL neg/AmpC neg	1	0 (0)		100		0 (0)		100		0 (0)		100	1/1 (100)				
All KPC (*n* = 47)	ESBL pos/AmpC neg	23	21 (91.3)	91.3			23 (100)	100			0 (0)		100	11/23 (47.8)				
	ESBL pos/pAmpC pos	1	1 (100%)	100			1	100			0 (0)			1/1 (100)				
	ESBL neg/AmpC neg	20	1 (5)		95		1 (5)		95		0 (0)		100	5/20 (25)				
	ESBL neg/gAmpC pos	3	0 (0)		100		0 (0)		100									
	Overall positive results		22/24 (91.7)	91.7			24/24 (100)	100			0/1 (0)	0						
	Overall negative results		22/23 (95.7)		95.7		22/23 (95.7)		95.7		27/43 (62.8)		62.8	17/43 (39.5)				
	Overall inconclusive													17/44 (38.6)				
IMP (*n* = 3)	ESBL neg/AmpC neg	3	1 (33.3)		66.7		0 (0)		100					3/3 (100)	0 (0)		100	
NDM (*n* = 49)	ESBL pos/AmpC neg	18	4 (23.5)	23.5		1/18 (5.5)	18 (100)	100			0 (0)	-	100	16/18 (88.8)	0 (0)		100	2/18 (11.1)
	ESBL pos/gAmpC pos	4	2 (50)	50			4 (100)	100										
	ESBL pos/pAmpC pos	9	1 (10)	10		1/9 (11.1)	9 (100)	100						9/9 (100)	1 (14.3)	14.3		2/9 (22.2)
	ESBL neg/AmpC neg	11	3 (27.3)		72.7		0 (0)		100					11/11 (100)	0 (0)		100	1/11 (9)
	ESBL neg/gAmpC pos	2	0 (0)		100		0 (0)		100									
	ESBL neg/pAmpC pos	5	0 (0)		100		0 (0)		100					5/5 (100)	2 (100)	100		3/5 (60)
NDM/OXA-48-type (*n* = 6)	ESBL pos/AmpC neg	2	0 (0)	0			2 (100)	100						2/2 (100)	0 (0)		100	
	ESBL pos/pAmpC pos	2	0 (0)	0			1 (50)	50						2/2 (100)	0 (0)	0		1/2 (50)
	ESBL neg/AmpC neg	1	0 (0)		100		0 (0)		100					1/1 (100)	0 (0)		100	1/1 (100)
	ESBL neg/gAmpC pos	1	0 (0)		100		0 (0)		100									
VIM (*n* = 12)	ESBL pos/gAmpC pos	4	0 (0)	0			4 (100)		100									
	ESBL neg/AmpC neg	4	0 (0)		100		0 (0)		100		0 (0)	-	100	3/4 (75)	0 (0)		100	
	ESBL neg/gAmpC pos	3	1 (33.3)		66.7		0 (0)		100									
	ESBL neg/pAmpC pos	1	0 (0)		100		0 (0)		100					1/1 (100)	1 (100)	100		
VIM/OXA-48-type (*n* = 1)	ESBL pos/AmpC neg	1	0 (0)	0			0 (0)	0			0 (0)		100		0 (0)		100	
All MBL (*n* = 71)	ESBL pos/AmpC neg	21	4 (20)	20		1/21 (4.8)	20 (95.2)	95.2			0 (0)		100	18/21 (85.7)	0 (0)		100	2/21 (9.5)
	ESBL pos/gAmpC pos	8	2 (25)	25			8 (100)	100										
	ESBL pos/pAmpC pos	11	1 (10)	10		1/10 (10)	10 (90.1)	90.1						11/11 (100)	1 (12.5)	12.5		3/11 (27.3)
	ESBL neg/AmpC neg	19	4 (21)		79		0 (0)		100		0 (0)		100	18/19 (94.7)	0 (0)		100	2/19 (10.5)
	ESBL neg/gAmpC pos	6	1 (16.6)		83.4		0 (0)		100									
	ESBL neg/pAmpC pos	6	0 (0)		100		0 (0)		100					6/6 (100)	3 (100)	100		3/6 (50)
	Overall positive results		7/40 (17.5)	17.5		2/40 (0.5)	38/40 (95)	95			0/17 (0)	0		17/17 (100)	4/17 (23.5)	23.5		6/17 (35.3)
	Overall negative results		26/31 (83.9)		83.9		31/31 (100)		100		4/40 (10)		10	36/40 (90)	36/40 (90)		90	4/40 (10)
	Overall inconclusive					2/71 (2.8)								53/57 (93)				10/57 (17.5)
NDM/KPC (*n* = 2)	ESBL neg/AmpC neg	2	0 (0)		100		0 (0)		100		0/2 (0)		0	2/2 (100)	0 (0)	-	100	1/2 (50)
VIM/KPC (*n* = 1)	ESBL pos/gAmpC pos	1	0 (0)	0			1 (100)	100										
All MBL/KPC (*n* = 3)	Overall positive results		0/1 (0)	0			1/1 (100)	100										
	Overall negative results		2/2 (100)		100		2/2 (100)	100	100		0/2		0		1/2 (50)		50	
	Overall inconclusive													2/2 (100)				1/2 (50)

a No., number, gAmpC, genomic AmpC; pAmpC, plasmidic AmpC.

**TABLE 3:** Performances of the confirmatory ESBL combination disc diffusion and MULTI-CTX-M tests

Method	Positive	False positive	Inconclusive	Negative	False negative
KPC (*n* = 47)					
ESBL confirmatory combination disc diffusion					
Standard	22/24 (91.7%)	1/23		22/23 (95.7%)	2/24
Modified	24/24 (100%)	1/23		22/23 (95.7%)	0/24
MULTI-CTX-M test^*a*^	23/23 (100%)	0/23		24/24 (100%)	0/24
MBL (*n* = 71)					
ESBL confirmatory combination disc diffusion					
Standard	7/40 (17.5%)	5/31	2/40 (0.5%)	26/31 (83.9%)	33/40
Modified	38/40 (95%)	0/31		31/31 (100%)	2/40
MULTI-CTX-M test^*b*^	39/39 (100%)	0/30		30/30 (100%)	0/39
MBL + KPC (*n* = 3)					
ESBL confirmatory combination disc diffusion					
Standard	0/1 (0%)	0/2		2/2 (100%)	1/1
Modified	1/1 (100%)	0/2		2/2 (100%)	0/1
MULTI-CTX-M test				3/3 (100%)	0/3

a Non-CTX-M ESBL-producing isolates were considered negatives.

b The MULTI CTX-M test was not performed with the 2 K. oxytoca isolates.

### Modified CDD results on KPC-producing CPE.

Within the KPC-producing isolates the modified CDD test allowed ESBL identification in all 24 ESBL-positive isolates (sensitivity, 100%) and, as for the standard CDD method, only one false-positive result was observed for the 23 ESBL-negative isolates (specificity, 95.7%). The corresponding isolate carried an inhibitor-resistant TEM (TEM-163) (10). Of note, the only SHV-type ESBL was phenotypically detected.

### Standard EUCAST CDD results on MBL CPE.

Regarding the 71 MBL producers, the standard CDD test allowed ESBL identification in only 7/40 ESBL-positive isolates (sensitivity, 17.5%) and generated 5 false-positive results among the 31 ESBL-negative isolates (specificity, 83.9%). Also, inconclusive results were produced with 2/40 ESBL-positive strains (both Klebsiella pneumoniae isolates producing NDM and a CTX-M-15). Regarding AmpC detection, inconclusive results were generated with all the plasmidic AmpC-positive (*n* = 17, 100%) and 36/40 (90%) AmpC-negative isolates, while no false-positive result was obtained with the remaining 4/40 plasmidic AmpC-negative strains (specificity, 10%).

### Modified CDD results on MBL.

Regarding the 71 MBL producers, the modified CDD test allowed ESBL detection in 38/40 ESBL-positive isolates (sensitivity, 95%), and no false-positive result was produced with the 31 ESBL-negative isolates (specificity, 100%). The two isolates where ESBL could not be phenotypically detected coproduced an MBL, an OXA-type carbapenemase, and a CTX-M-type ESBL (see Table S1 in the supplemental material). Regarding AmpC detection, inconclusive results were generated with 6/17 (35.3%) plasmidic AmpC-positive and 4/40 (10%) AmpC-negative isolates. AmpC production was confirmed in 4/17 plasmidic AmpC-positive strains (3/14 CMY- and 1/3 DHA-type AmpC, sensitivity, 23.5%) and excluded in 36/40 plasmidic AmpC-negative isolates (specificity, 90%).

### Standard and modified EUCAST CDD results on KPC/MBL CPE.

Of the 3 KPC/MBL producers, using the standard CDD test, the ESBL was not detected in the only PER-type ESBL producer and no false-positive result was produced with the 2 ESBL-negative isolates. Also, regarding AmpC detection, inconclusive results were obtained with the two AmpC-negative strains.

Among the 3 KPC/MBL coproducing isolates, using the modified CDD test, the ESBL was correctly detected in the only ESBL producer (PER type) and not detected in the 2 ESBL-negative isolates. Regarding AmpC detection, inconclusive results were obtained with one AmpC-negative isolate and AmpC was not detected in the other.

### Increases in the inhibition zone diameters using the standard and modified EUCAST CDD on KPC/MBL CPE.

Among class A CPE isolates, for 20 ESBL-only producers, the modified method showed higher increases in inhibition zone diameters (induced by clavulanic acid) of disc-containing cefotaxime (mean of 11 mm versus 13 mm with the standard and modified method, respectively) and ceftazidime (mean of 6.1 mm versus 7.3 mm with the standard and modified method, respectively) compared to the standard method (Table 4). This effect was much more pronounced among class B CPE isolates, where for 18 NDM/ESBL-only producers the mean differences of cefotaxime and ceftazidime with and without clavulanic acid were 4 mm and 3 mm with the standard and 15.5 mm and 9.9 mm with the modified method, respectively.

**TABLE 4:** Average increases in growth inhibition diameters of cefotaxime, ceftazidime ± clavulanic acid and cefoxitin ± cloxacillin by the standard and modified EUCAST ESBL confirmatory disc diffusion tests^*a*^

Phenotypic confirmatory test			ESBL detection				AmpC detection	
			Standard		Modified		Standard	Modified
Genotype			CTX/CA-CTX	CAZ/CA-CAZ	CTX/CA-CTX	CAZ/CA-CAZ	FOX/CLO-FOX	FOX/CLO-FOX
Carbapenemase(s)	ESBL/AmpC	No.						
KPC	CTX-M type/-	20	11	6.1	13	7.3		
	SHV type/-	1	8	11	7	14		
	CTX-M/SHV type/-	1	4	4	9	11		
	CTX-M/SHV type/CMY type pAmpC	1	10	10	11	11	0	0
KPC/OXA-48-type	CTX-M type/-	1	2	4	9	11		
NDM	CTX-M type/-	18	4	3	15.5	9.9		
	CTX-M type/CMY type pAmpC	8	2.3	2.3	9.9	7.8	0	1.5
	CTX-M type/DHA type pAmpC	1	0	3	8	8	0	0
	CTX-M type/gAmpC	4	1.5	1.5	14	9.5		
	-/CMY type pAmpC	4					0	2.5
	-/DHA type pAmpC	1					0	9
NDM/OXA-48-type	CTX-M type/-	2	0	0	9	8.5		
	CTX-M type/CMY type pAmpC	1	0	0	0	4	0	0
	CTX-M type/DHA type pAmpC	1	0	0	9	1	0	2
VIM	CTX-M type/gAmpC	4	1	1.8	8.8	16		
	-/CMY type pAmpC	1					0	4
VIM/OXA-48-type	CTX-M type/-	1	0	0	4	1		
KPC/VIM	PER type/CMY type pAmpC	1	0	0	12	15	0	3

a CTX, cefotaxime; CTX/CA, cefotaxime/clavulanic acid; CAZ, ceftazidime; CAZ/CA, ceftazidime/calvulanic acid; FOX, cefoxitin; FOX/CLO, cefoxitin/cloxacilline.

### Performance of the CTX-M MULTI lateral flow immunoassay.

Regarding the 47 KPC producers, the CTX-M-MULTI lateral flow immunoassay resulted in a positive result for all 23 CTX-M-ESBL producers (sensitivity, 100%), while the 1 SHV-type ESBL-harboring isolate as well as all the 23 ESBL-negative strains gave negative results (specificity, 100%). With respect to the 71 MBL producers, the CTX-M-MULTI detected ESBLs in all 39 CTX-M-ESBL-producers (sensitivity, 100%), while all the 30 ESBL-negative isolates were correctly tested as negative. Finally, the 3 CTX-M-type ESBL-negative KPC/MBL producers gave negative results (specificity, 100%). Overall, the CTX-M-MULTI test displayed excellent analytical performance for CTX-M-ESBL detection with sensitivity and specificity both of 100%.

## DISCUSSION

The EUCAST confirmatory CDD test allows accurate detection of ESBL and AmpC in ESBL-only, AmpC-only, and ESBL/AmpC-coproducing *Enterobacterales* (9, 11). However, both sensitivity and specificity of this test can be significantly hampered when class A or class B carbapenemases are coproduced, since they can effectively hydrolyze the same substrates as ESBLs and AmpC, yet they are insensitive β-lactamase inhibitors, like clavulanic acid or cloxacillin (12).

In the present work we have confirmed that the modified CDD test allows a more robust detection of ESBL production in class A CPE (increased difference in growth inhibition zones between cefotaxime and ceftazidime ± clavulanic acid, leading to a sensitivity of 100% and specificity of 95.7%, comparable to those obtained by Poulou et al. [5], both 100%) compared to the EUCAST CDD standard method (91.7% sensitivity and 95.7% specificity). In class B CPE the modified CDD test also substantially improved ESBL identification (sensitivity and specificity, 95% and 100%, respectively, similar to those obtained by Poulou et al. [5], 97% and 100%, respectively) compared to the conventional method, which performed very poorly (17.5% sensitivity and 83.9% specificity). Furthermore, by using the same principle (use of inhibitors to block carbapenemase activity), we showed that the modified CDD improved the robustness in AmpC detection in MBL CPE. In fact, inconclusive results were produced for only 10/57 isolates (17.5%), compared to 53/57 (93%) obtained with the standard CDD method. This finding is consistent with the notion that MBLs can hydrolyze cefoxitin and their activity is not inhibited by cloxacillin (13, 14). The sensitivity, however, remained unsatisfactory (23.5%). In accordance with a previous study conducted by Bernabeu et al. (7), we have also shown that the CTX-M-MULTI test can accurately detect CTX-M-type ESBLs in class A and class B CPE (100% sensitivity and specificity), except for Klebsiella oxytoca. Among the 64 class A and class B CPE isolates coproducing an ESBL, only 2 harbored a non-CTX-M ESBL. Thus, in areas with a high prevalence of CTX-M-type ESBLs, the CTX-M-MULTI test may be performed first upon suspicion of ESBL production in class A and class B CPE, also considering the moderate cost of the assay (15 to 20 euros), and most importantly the very short turnaround time (20 min versus 24 h of the CDD confirmatory test), which may save considerable labor costs as well as generate and report the results in less than 30 min.

**FIG 1:**
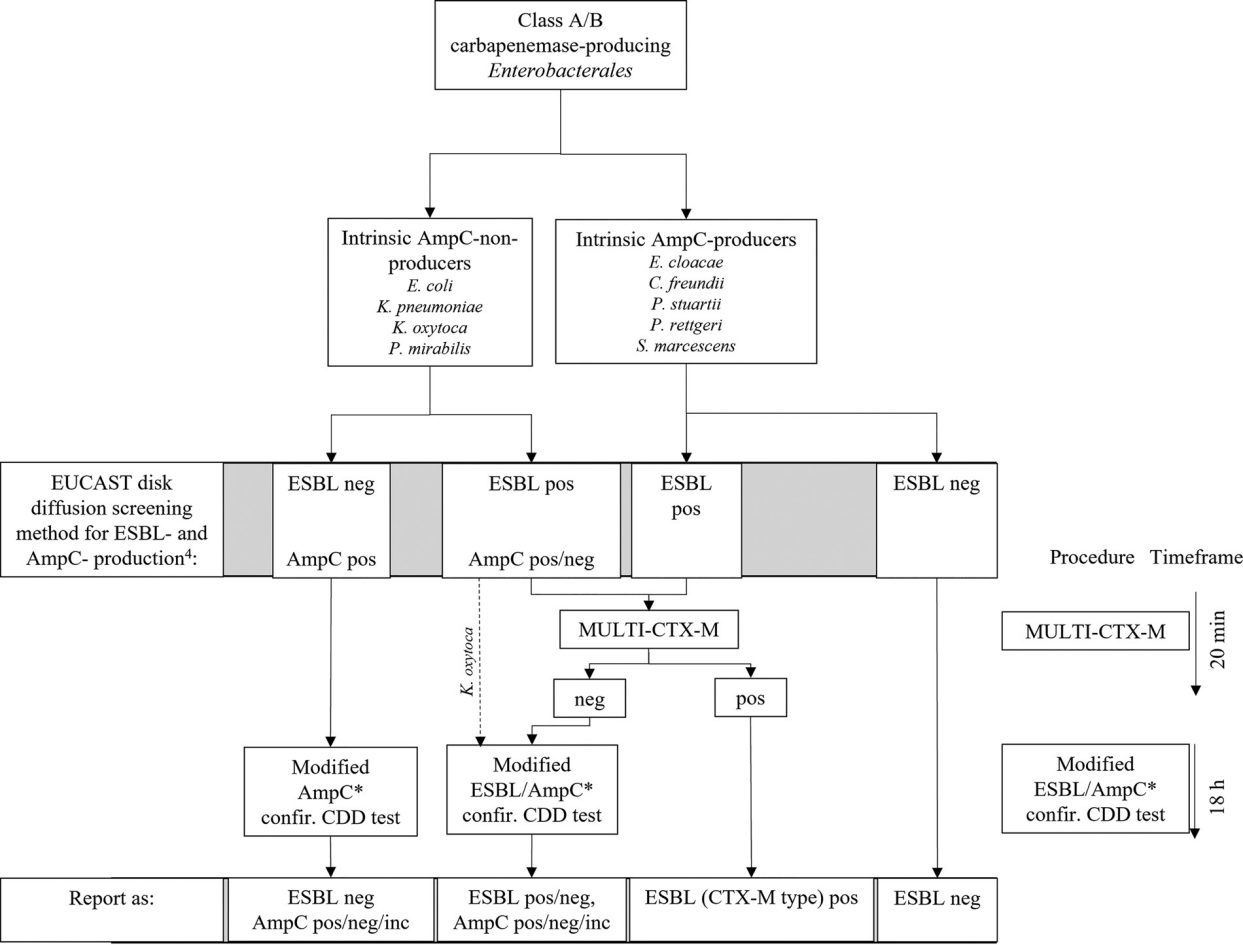
Diagnostic flow chart. Diagnostic approach combining the modified ESBL/AmpC confirmatory combined disc diffusion test and the CTX-M-MULTI test for confirmation of ESBL/AmpC production in class A- and class B-producing CPE. Pos, positive; neg, negative; inc, inconclusive; n.d., not determined; LFIA, lateral flow immunoassay. *The modified AmpC confirmatory CDD test should be performed only with class B CPE.

In view of the results presented here, we propose a practical diagnostic approach that combines the modified ESBL/AmpC confirmatory CDD test and the CTX-M-MULTI test for confirmation of ESBL/AmpC production in class A- and class B-producing CPE (Fig. 1). Except for K. oxytoca, the CTX-M-MULTI test may be performed with CPE isolates that do not produce an intrinsic AmpC and are suspected to harbor an ESBL, irrespective of whether they also may produce a plasmidic AmpC. If the result for ESBL is positive, AmpC production may be disregarded since the sensitivity of the phenotypic CDD test for AmpC detection is poor. If the CTX-M-MULTI test is negative, the modified CDD test can then be performed to confirm or rule out the presence of a non-CTX-M ESBL. For CPE strains not suspected to produce an ESBL, the modified CDD test may be performed (only with class B CPE) for confirmation of suspected AmpC production. Furthermore, K. oxytoca CPE isolates suspected to produce an ESBL and/or AmpC may be subjected to the modified CDD test, since for these strains the CTX-M-MULTI test is unreliable due to production of an intrinsic OXY-1 β-lactamase, which may give rise to false-positive results (15). Strains of species producing an intrinsic AmpC may also be initially examined using the CTX-M-MULTI test when ESBL production is suspected. In case of a negative result, the combined CDD test may then be carried out to confirm or exclude the presence of non-CTX-M ESBLs.

Although molecular assays (homemade or automated PCRs or WGS) may conceivably provide more accurate results in the identification of ESBL and/or AmpC genes, due to the low accessibility (especially in low-income countries) and comparably higher costs than the phenotypic and immunological methods (including the reagents, devices, labor), the proposed diagnostic algorithm is far more suitable for a clinical microbiology lab, especially in labs where disk diffusion is the standard antimicrobial susceptibility testing (AST).

In summary, we propose a practical and cost-effective approach for confirmation of ESBL and/or AmpC production in class A and class B CPE that is based on phenotypic and immunological tests. This diagnostic flow chart is accurate, cost-effective and, therefore, applicable in a standard routine clinical microbiology laboratory.

## MATERIALS AND METHODS

### Clinical isolates.

A total of 121 nonduplicate *Enterobacterales* clinical strains producing class A (KPC-type) and/or class B metallocarbapenemases (NDM, VIM, and IMP type) isolated between January 2011 and December 2022 in the routine diagnostic of the Institute of Medical Microbiology, University of Zurich, were included in this study (Table 1). Clinical isolates producing the same carbapenemase type that were derived from the same patient were considered duplicates and were thus excluded. The collection consisted of 76 K. pneumoniae, 24 E. coli, 8 E. cloacae, 6 C. freundii, 2 K. oxytoca, 2 P. stuartii, 1 P. mirabilis, 1 S. marcescens, and 1 P. rettgeri.

### MALDI-TOF MS identification.

Bacterial isolates were prepared for matrix-assisted laser desorption ionization–time of flight mass spectrometry (MALDI-TOF MS) by the direct transfer-formic acid method (16). Species identification was performed using the Bruker Biotyper MALDI-TOF MS System (Bruker Corporation, MA, USA).

### Screening and confirmation carbapenemase production.

AST was performed by the Kirby-Bauer diffusion method according to EUCAST guidelines (17). The inhibition zone diameters were measured with the Sirweb/Sirscan system (i2a) (18). Screening for carbapenemase production was performed using the EUCAST screening cutoff values for carbapenemase-producing *Enterobacterales* (4). Carbapenemase production was confirmed by CDD based on the diagnostic flow chart elaborated by Maurer et al. (19). Meropenem (10 μg) and ertapenem (10 μg) discs (Becton, Dickinson; Franklin Lakes, NJ, USA) alone or in combination with 10 μL of a 30-mg/mL amino phenylboronic acid (APBA) solution or 10 μL of 0.1 M Na-EDTA solution, respectively, were used to confirm the presence of class A and B carbapenemases. Differences in inhibition growth diameters of ≥ 5 mm between the APBA-free and APBA-containing discs or between the EDTA-free and EDTA-containing discs were considered indicative of class A and B carbapenemase production, respectively. Temocillin (10 μg) discs (Becton, Dickinson) on Mueller-Hinton cloxacillin agar plates (Axonlab AG, Baden, Switzerland) were used to confirm the presence of class D (OXA-type) carbapenemases. Temocillin zone diameters smaller than 11 mm were considered indicative of class D carbapenemase production. Unclear results were resolved either with the AID carbapenemase line probe assay (Autoimmun Diagnostika GmbH, Straßberg, Germany) (20) or with the Carba-5 lateral-flow immunoassay (NG Biotech, Guipry, France) (21).

### Screening and confirmation of ESBLs and AmpCs.

Screening for ESBLs and AmpCs was performed by disc diffusion according to the EUCAST guidelines for detection of resistance mechanisms and specific resistances of clinical and/or epidemiological importance (4). ESBL production in AmpC-negative isolates was confirmed by the EUCAST CDD test (4) on Mueller-Hinton agar plates (bioMérieux, Marcyl’Etoile, France) based on the diagnostic flow chart elaborated by Polsfuss et al. (9) using both cefotaxime (30 mg) and ceftazidime (30 mg) discs (Becton, Dickinson) alone or in combination with clavulanic acid (10 mg). The results are reported on Table S1. The test was considered positive when the inhibition zone diameter of any of the two cephalosporin/clavulanic acid discs was increased by ≥ 5 mm compared to the corresponding disc without clavulanic acid. The test was considered inconclusive when only one test was marginally positive (difference between the growth inhibition zone ± clavulanic acid = 5 mm). For K. oxytoca only, ceftazidime ± clavulanic acid was considered. For AmpC-producing isolates (intrinsic or acquired as determined by the AmpC confirmatory CDD test, see below) the CDD test for ESBL detection was performed on Mueller-Hinton cloxacillin agar plates (Axonlab AG). Acquired AmpC production (or over induction of the intrinsic *ampC* in E. coli isolates) was confirmed in E. coli isolates and strains without a genomic *ampC* gene (K. pneumoniae, K. oxytoca and P. mirabilis) by the CDD test as described by Polsfuss et al. (11) using cefoxitin (30 mg) discs alone or in combination with cloxacillin (200 μg) (Liofilchem, Roseto degli Abruzzi, Italy). The test was considered positive when the inhibition zone diameter of the cefoxitin/cloxacillin disc was increased by ≥4 mm compared to the disc containing only cefoxitin. The test was considered inconclusive when no inhibition zones (≤6 mm) around both cefoxitin discs with and without cloxacillin were detected. Based on the algorithm, inconclusive results should be resolved by means of another method.

The modified method for detection of ESBL was performed as described by Poulou et al. (5). The modified CDD confirmatory method for AmpC was performed following the same principle (use of carbapenemase inhibitors to block their activity) only on MBL producers, since APBA is a reversible inhibitor of AmpC β-lactamases. The results are reported on Table S1. Briefly, 10 μL of 0.1 M Na-EDTA solution (for *Enterobacterales*-producing class B MBLs) were added to all the discs used in the standard method, and 10 μL of 40 mg/mL APBA (for *Enterobacterales*-producing class A KPC-type carbapenemases) solution were added to discs containing ceftazidime and cefotaxime ± clavulanic acid (but not cefoxitin ± cloxacillin). For isolates coproducing class A and class B carbapenemases, 10 μL of both solutions were added to discs containing ceftazidime and cefotaxime ± clavulanic acid, while only 10 μL of the Na-EDTA solution was added to discs with cefoxitin ± cloxacillin. Results were interpreted in the same way as for the standard method.

### Immunochromatographic detection of CTX-M-type ESBLs.

Production of CTX-M-type ESBLs was confirmed with the CTX-M-MULTI (V2) lateral-flow immunoassay according to the manufacturer’s instructions (NG Biotech) (7). Due to known possible cross-reactivity of intrinsic OXY-1-type β-lactamases with monoclonal antibodies contained in the CTX-M-MULTI assay, this test was not performed with K. oxytoca isolates (15).

### Whole-genome sequencing.

DNA was extracted from the bacterial isolates using the DNeasy Ultraclean microbial kit (Qiagen, Hilden, Germany) according to the manufacturer’s instructions. Library preparation was done with the QIASeq FX kit (Qiagen). Library quality and fragment size distribution was analyzed by capillary electrophoresis on a fragment analyzer automated CE system (Advanced Analytical Technologies Inc., Heidelberg, Germany). DNA libraries were pooled in equimolar concentrations and paired-end sequencing (2 × 150 bp) was performed using an Illumina MiSeq platform (Illumina, San Diego, CA, USA).

### Detection of β-lactamase resistance genes.

Raw sequencing data were filtered and trimmed with Trimmomatic (version 0.39) (22). Reads were further assembled using SPAdes (23). Plasmidic β-lactamase genes and chromosomal mutations in E. coli chromosomal *bla*_ampC_ promoter/attenuator regions were identified querying CARD (24, 25) and/or using in-house analysis pipelines.
