# Supplementary material for: Detection of Extended-Spectrum β-Lactamases (ESBLs) and AmpC in Class A and Class B Carbapenemase-Producing Enterobacterales
Source: Microbiol Spectr. 2022 Oct 26;10(6):e02137-22. doi: 10.1128/spectrum.02137-22 (PMC9769508; doi:10.1128/spectrum.02137-22)
Supplement: Supplemental file 1 — Table S1. Download spectrum.02137-22-s0001.pdf, PDF file, 0.7 MB [file spectrum.02137-22-s0001.pdf]

Table S1. β-lactamase content and results of the combination disc diffusion test of all the clinical strains used in this study

MBL-producers							Standard method															Modified method														
							MH agar plate									MH-cloxacillin plate						MH agar plate									MH-cloxacillin plate					
No.	Strain	Carbapenemase(s)	AmpC	ESBL	Other β-lactamase(s)	Multi CTX-M	FOX/CLOXA	FOX	FOX/CLOXA - FOX	CTX/CLA	CTX	CTX/CLA-CTX	CAZ/CLA	CAZ	CAZ/CLA - CAZ	CTX/CLA	CTX	CTX/CLA-CTX	CAZ/CLA	CAZ	CAZ/CLA - CAZ	FOX/CLOXA	FOX	FOX/CLOXA - FOX	CTX/CLA	CTX	CTX/CLA-CTX	CAZ/CLA	CAZ	CAZ/CLA - CAZ	CTX/CLA	CTX	CTX/CLA-CTX	CAZ/CLA	CAZ	CAZ/CLA - CAZ
5	*E. coli*	IMP-1	-	-	TEM-1	-	6	6	0	14	12	2	12	6	6	13	13	0	12		12	21	21	0	25	27	-2	25	22	3	27	27	0	25	22	3
43	*E. coli*	IMP-1	-	-	TEM-1	-	6	6	0	8	9	-1	9	6	3	12	13	-1	12	6	6	19	20	-1	25	23	2	24	23	1	25	26	-1	26	22	4
94	*K. pneumoniae*	IMP-1	-	-	SHV-11/TEM-1	-	6	6	0	13	13	0	9	6	3	12	11	1	11	6	5	20	20	0	25	25	0	24	24	0	24	23	1	23	22	1
36	*C. freundii*	NDM-1	CMY	-	TEM-1	-	6	6	0	6	6	0	6	6	0	7	6	1	6	6	0	11	6	5	13	13	0	15	11	4	20	19	1	18	15	3
117	*C. freundii*	NDM-1	CMY	-	TEM-1/OXA-1	-	6	6	0	9	8	1	6	6	0	11	10	1	8	6	2	19	9	10	30	29	1	27	26	1	28	31	-3	30	26	4
147	*E. cloacae*	NDM-1	ACT-25/CMY-4	CTX-M-15	TEM-1/OXA-1	+	6	6	0	6	6	0	6	6	0	6	6	0	6	6	0	8	6	2	8	6	2	11	7	4	16	6	10	19	6	13
172	*E. cloacae*	NDM-1	-	CTX-M-15	OXA-9/OXA-140/OXA-299/TEM-1	+	6	6	0	6	6	0	6	6	0	6	6	0	6	6	0	6	6	0	17	6	11	14	6	8	26	6	20	24	12	12
67	*E. coli*	NDM-1	-	-	OXA-1/OXA-10/TEM-1	-	6	6	0	9	6	3	12	6	6	9	6	3	6	6	0	17	17	0	19	17	2	16	14	2	26	26	0	27	23	4
71	*E. coli*	NDM-1	-	-	OXA-1/TEM-1	-	6	6	0	14	13	1	13	6	7	14	14	0	10	6	4	20	22	-2	23	24	-1	20	18	2	32	33	-1	30	28	2
124	*E. coli*	NDM-1	CM	-	-	-	6	6	0	6	6	0	6	6	0	6	6	0	10	6	4	19	9	10	13	13	0	17	13	4	28	28	0	27	25	2

			Y-6																																	
171	*E. coli*	NDM-1	-	CTX-M-15	TEM-192	+	6	6	0	10	6	4	6	6	0	10	6	4	8	6	2	16	18	-2	18	12	6	18	16	2	27	15	12	23	20	3
51	*K. pneumoniae*	NDM-1	-	-	SHV-11	-	6	6	0	9	6	3	10	6	4	8	6	2	12	6	6	17	15	2	22	22	0	20	19	1	24	25	-1	20	21	-1
82	*K. pneumoniae*	NDM-1	-	-	SHV-11	-	6	6	0	9	6	3	12	6	6	10	6	4	12	6	6	13	12	1	22	24	-2	22	19	3	23	24	-1	22	22	0
179	*K. pneumoniae*	NDM-1	-	-	OKP-B-19	-	6	6	0	12	11	1	10	6	4	15	13	2	12	6	6	22	23	-1	26	28	-2	24	21	3	26	28	-2	24	23	1
138	*K. pneumoniae*	NDM-1	CMY-4	CTX-M-15	SHV-1/OXA-1	+	6	6	0	6	6	0	6	6	0	6	6	0	6	6	0	14	11	3	22	6	16	20	11	9	24	9	15	22	10	12
103	*K. pneumoniae*	NDM-1	-	CTX-M-15	OXA-1	+	6	6	0	9	6	3	8	6	2	9	6	3	10	6	4	23	23	0	25	6	19	25	11	14	25	7	18	25	12	13
8	*K. pneumoniae*	NDM-1	-	CTX-M-15	SHV-1/TEM-1/OXA-1	+	6	6	0	11	6	5	11	6	5	13	6	7	12	6	6	25	26	-1	25	6	19	26	13	13	25	7	18	29	12	17
24	*K. pneumoniae*	NDM-1	-	CTX-M-15	SHV-11/OXA-9	+	6	6	0	6	6	0	6	6	0	6	6	0	10	6	4	22	22	0	24	6	18	25	13	12	23	8	15	22	13	9
48	*K. pneumoniae*	NDM-1	-	CTX-M-15	SHV-28/OXA-9/TEM-1	+	6	6	0	10	6	4	9	6	3	10	6	4	6	6	0	19	21	-2	23	6	17	22	12	10	23	7	16	21	12	9
52	*K. pneumoniae*	NDM-1	-	CTX-M-15	SHV-11/OXA-1	+	6	6	0	10	6	4	10	6	4	11	6	5	11	6	5	19	19	0	23	6	17	22	11	11	23	6	17	22	11	11
54	*K. pneumoniae*	NDM-1	-	CTX-M-15	SHV-28/OXA-9/TEM-1	+	6	6	0	10	6	4	10	6	4	12	6	6	13	6	7	17	17	0	20	6	14	16	11	5	25	11	14	24	6	18
100	*K. pne*	NDM-1	-	CTX-M-15	SHV-28/OXA-9/TEM-1	+	6	6	0	10	6	4	10	6	4	9	6	3	11	6	5	21	21	0	24	6	18	25	12	13	24	6	18	23	12	11

	umoniae																																			
106	*K. pneumoniae*	NDM-1	-	CTX-M-15	SHV-11/OXA-1/TEM-1	+	13	13	0	25	6	19	24	10	14	24	6	18	22	6	16	14	14	0	22	6	16	25	6	19	22	6	16	26	6	20
174	*K. pneumoniae*	NDM-1	-	CTX-M-15	SHV-11/OXA-9/OXA-140/TEM-118	+	6	6	0	9	6	3	6	6	0	10	6	4	6	6	0	18	20	-2	21	6	15	17	6	11	25	8	17	24	8	16
185	*K. pneumoniae*	NDM-1	-	CTX-M-15	SHV-11/OXA-9/OXA-140/TEM-118	+	6	6	0	9	6	3	6	6	0	10	6	4	6	6	0	22	23	-1	22	6	16	21	6	15	32	10	22	30	13	17
187	*K. pneumoniae*	NDM-1	-	CTX-M-15	SHV-11/OXA-9/OXA-140/TEM-118	+	6	6	0	10	6	4	6	6	0	10	6	4	6	6	0	24	23	1	23	6	17	18	6	12	28	11	17	28	11	17
28	*K. pneumoniae*	NDM-1	CMY-16	CTX-M-15	SHV-28/OXA-10/OXA-1/TEM-1	+	6	6	0	9	6	3	8	6	2	11	6	5	9	6	3	13	11	2	16	9	7	16	6	10	21	11	10	18	11	7
29	*K. pneumoniae*	NDM-1	CMY-16	CTX-M-15	SHV-28/OXA-10/OXA-1/TEM-1	+	6	6	0	6	6	0	9	6	3	9	6	3	8	6	2	11	12	-1	19	9	10	17	10	7	18	10	8	18	10	8
80	*K. pneumoniae*	NDM-1	CMY-16	CTX-M-15	SHV-28/OXA-1/OXA-10/TEM-1	+	6	6	0	11	6	5	9	6	3	12	6	6	11	6	5	11	11	0	16	8	8	11	6	5	19	6	13	17	10	7
101	*K. pneumoniae*	NDM-1	CMY-16	CTX-M-15	SHV-28/OXA-1/OXA-10/TEM-1	+	6	6	0	11	8	3	10	6	4	10	6	4	9	6	3	14	13	1	21	11	10	19	12	7	25	11	14	18	13	5
41	*K. pneumoniae*	NDM-1	CMY-6	CTX-M-15	SHV-11/OXA-1/TEM-1	+	6	6	0	10	6	4	10	6	4	6	6	0	11	6	5	8	6	2	15	6	9	18	9	9	22	6	16	25	9	16
192	*K. pneumoniae*	NDM-1	DHA-1	CTX-M-15	SHV-28/OXA-1/TEM-1	+	6	6	0	6	6	0	9	6	3	8	6	2	6	6	0	6	6	0	14	6	8	14	6	8	28	11	17	24	11	13
128	*K. pne*	NDM-1		CTX-M-15	SHV-11/OXA-9/TEM-1	+	6	6	0	9	6	3	11	6	5	9	6	3	12	6	6	19	20	-1	27	6	21	24	11	13	26	10	16	24	10	14

	umoniae																																			
194	*P. mirabilis*	NDM-1	-	CTX-M-2/CTX-M-8	OXA-2/OXA-10/TEM-1	+	9	9	0	14	6	8	15	6	9	32	29	3	34	17	17	26	26	0	31	6	25	28	25	3	34	6	28	37	34	3
188	*P. stuartii*	NDM-1	-	CTX-M-2/CTX-M-8	OXA-2/OXA-10/TEM-1	+	16	15	1	14	12	2	12	6	6	19	16	3	16	6	10	25	26	-1	28	18	10	26	21	5	32	19	13	33	27	6
193	*P. stuartii*	NDM-1	-	CTX-M-2/CTX-M-8	OXA-2/OXA-10/TEM-1	+	15	16	-1	14	10	4	6	6	0	19	16	3	14	6	8	28	28	0	33	16	17	29	27	2	32	19	13	33	26	7
127	*E. coli*	NDM-19	-	-	-	-	6	6	0	6	6	0	6	6	0	6	6	0	6	6	0	11	9	2	20	19	1	22	20	2	21	21	0	23	20	3
37	*K. pneumoniae*	NDM-4	CMY-6	CTX-M-15	TEM-1	+	6	6	0	9	6	3	6	6	0	10	6	4	9	6	3	17	12	5	17	6	11	16	6	10	23	10	13	21	11	10
123	*E. coli*	NDM-5	-	-	TEM-1	-	6	6	0	10	6	4	9	6	3	9	6	3	8	6	2	26	26	0	26	26	0	24	21	3	27	27	0	28	24	4
125	*E. coli*	NDM-5	-	-	TEM-1	-	6	6	0	6	6	0	8	6	2	6	6	0	10	6	4	9	7	2	19	19	0	20	18	2	19	19	0	19	16	3
160	*E. coli*	NDM-5	-	-	-	-	6	6	0	6	6	0	6	6	0	6	6	0	6	6	0	6	6	0	12	12	0	10	11	-1	19	21	-2	19	18	1
191	*E. coli*	NDM-5	CMY-42	-	-	-	6	6	0	6	6	0	6	6	0	6	6	0	6	6	0	6	6	0	6	6	0	6	6	0	6	6	0	9	6	3
4	*E. coli*	NDM-5	CMY-42	-	TEM-1	-	6	6	0	6	6	0	6	6	0	6	6	0	6	6	0	6	6	0	20	18	2	20	18	2	19	20	-1	20	18	2
157	*E. coli*	NDM-5	CMY-42	-	TEM-1	-	6	6	0	6	6	0	6	6	0	6	6	0	6	6	0	6	6	0	6	6	0	6	6	0	14	14	0	18	19	-1
18	*E. coli*	NDM-5	-	CTX-M-15	TEM-1	+	6	6	0	6	6	0	6	6	0	6	6	0	6	6	0	17	17	0	21	6	15	21	6	15	21	6	15	19	6	13
66	*E. coli*	NDM-5	-	CTX-M-15	OXA-1/TEM-1	+	6	6	0	6	6	0	6	6	0	6	6	0	6	6	0	6	6	0	12	6	6	13	10	3	21	6	15	20	14	6

186	*E. coli*	NDM-5	-	CTX-M-15	OXA-1	+	6	6	0	10	6	4	10	6	4	10	6	4	13	6	7	6	6	0	13	6	7	13	6	7	20	6	14	21	10	11
165	*E. coli*	NDM-5	CMY-2	CTX-M-15	OXA-1/TEM-1	+	6	6	0	6	6	0	8	6	2	9	6	3	12	6	6	6	6	0	12	6	6	11	6	5	16	6	10	19	6	13
112	*E. coli*	NDM-5	-	CTX-M-24	TEM-1	+	6	6	0	6	6	0	6	6	0	6	6	0	6	6	0	14	13	1	19	6	13	18	17	1	18	6	12	19	15	4
55	*K. pneumoniae*	NDM-5	-	-	SHV-1/TEM-1	-	6	6	0	9	6	3	8	6	2	6	6	0	6	6	0	17	18	-1	26	26	0	24	23	1	25	26	-1	23	22	1
156	*K. pneumoniae*	NDM-5	DHA-1	-	SHV-11	-	6	6	0	11	9	2	6	6	0	11	9	2	6	6	0	15	6	9	18	24	-6	19	21	-2	26	25	1	22	22	0
122	*E. coli*	NDM-7	-	-	-	-	6	6	0	6	6	0	8	6	2	6	6	0	6	6	0	12	10	2	19	21	-2	21	19	2	21	22	-1	23	20	3
207	*K. oxytoca*	OXA-48/VIM-1		CTX-M-9	OXY-1-1	N.p.	8	9	1	10	10	0	6	6	0	12	12	0	6	6	0	16	15	1	20	16	4	24	23	1	21	17	4	23	23	0
166	*K. pneumoniae*	OXA-181/NDM-4	DHA-1	CTX-M-15	SHV-11/OXA-1/TEM-1	+	6	6	0	6	6	0	6	6	0	6	6	0	6	6	0	8	6	2	15	6	9	10	9	1	20	10	10	19	14	5
184	*E. coli*	OXA-244/NDM-5	-	-	-	-	6	6	0	6	6	0	6	6	0	6	6	0	6	6	0	6	6	0	6	10	-4	10	9	1	18	18	0	19	19	0
154	*E. cloacae*	OXA-48/NDM-1	ACT-32	-	TEM-1/OXA-1	-	6	6	0	6	6	0	6	6	0	6	6	0	6	6	0	6	6	0	25	27	-2	26	23	3	24	26	-2	23	24	-1
159	*K. pneumoniae*	OXA-48/NDM-1	-	CTX-M-15	SHV-11/OXA-9/TEM-1	+	6	6	0	6	6	0	6	6	0	6	6	0	6	6	0	12	11	1	12	6	6	15	6	9	15	6	9	19	9	10
202	*K. pne*	OXA-48/N	-	CTX-M-15	OXA-9/SHV-11	+	6	6	0	6	6	0	6	6	0	6	6	0	6	6	0	10	10	0	18	6	12	14	6	8	15	6	9	20	10	10

	umoniae	DM-1																																		
200	*K. pneumoniae*	OXA-48/NDM-1	CMY-6	CTX-M-15	SHV-28	+	6	6	0	6	6	0	6	6	0	6	6	-6	6	6	0	6	6	0	6	6	0	10	6	4	9	6	3	15	8	7
25	*C. freundii*	VIM-1	CMY-65	-	OXA-1	-	11	6	5	10	12	-2	12	6	6	14	14	0	10	6	4	23	9	14	28	30	-2	25	24	1	28	28	0	26	27	-1
3	*E. cloacae*	VIM-1	ACT-20	-		-	6	6	0	6	6	0	10	6	4	13	11	2	12	6	6	8	6	2	6	6	0	10	6	4	20	22	-2	24	23	1
126	*E. cloacae*	VIM-1	ACT-17	CTX-M-9	SHV-66	+	6	6	0	6	6	0	7	6	1	10	9	1	9	6	3	9	6	3	25	16	9	25	13	12	24	16	8	26	6	20
15	*E. cloacae*	VIM-1	ACT	CTX-M-9/SHV-12	-	+	6	6	0	6	6	0	9	6	3	6	6	0	6	6	0	6	6	0	17	6	11	23	6	17	17	6	11	23	6	17
16	*E. cloacae*	VIM-1	ACT	CTX-M-9/SHV-12	-	+	6	6	0	6	8	-2	9	6	3	9	6	3	10	6	4	8	6	2	26	17	9	25	14	11	25	18	7	26	15	11
17	*E. cloacae*	VIM-1	ACT	CTX-M-9/SHV-12	-	+	6	6	0	6	6	0	6	6	0	6	6	0	6	6	0	6	6	0	17	6	11	11	6	5	15	6	9	22	6	16
60	*K. oxytoca*	VIM-1	-	-	TEM-1/OXY-2-8	N.p.	6	6	0	12	12	0	10	6	4	11	9	2	9	6	3	15	14	1	20	18	2	18	18	0	24	22	2	25	22	3
9	*K. pneumoniae*	VIM-1	-	-	SHV-11	-	6	6	0	6	6	0	10	6	4	6	6	0	6	6	0	14	15	-1	26	25	1	23	21	2	24	27	-3	25	23	2
68	*K. pneumoniae*	VIM-1	-	-	SHV-11	-	6	6	0	11	10	1	10	6	4	11	11	0	9	6	3	17	16	1	20	22	-2	19	17	2	30	30	0	24	22	2
114	*K. pneumoniae*	VIM-1	-	-	OKP-A-11	-	18	17	1	18	17	1	14	12	2	19	18	1	13	11	2	24	25	-1	29	30	-1	26	25	1	30	29	1	26	27	-1
76	*K. pne*	VIM-1	CM	-	SHV-1	-	6	6	0	9	9	0	6	6	0	10	10	0	6	6	0	10	6	4	15	19	-4	11	16	-5	28	30	-2	24	24	0

	umoniae		Y-13																														
58	*S. marcescens*	VIM-5	-	-	OXA-2/SRT	-	15	12	3	9	10	-1	16	16	0	10	11	-1	16	16	0	12	10	2	17	16	1	22	21	1	26	25	1	29	26	3

KPC-producers

151	*C. freundii*	KPC-2	CMY-113	-	TEM-163?	-	13	10	3	25	26	-1	25	25	0	23	25	-2	24	23	1	12	12	0	25	24	1	23	22	1	25	26	-1	24	24	0
182	*C. freundii*	KPC-2	CMY	-	TEM-1	-	11	11	0	20	22	-2	20	19	1	21	23	-2	21	21	0	10	6	4	22	21	1	19	19	0	21	22	-1	21	19	2
198	*C. freundii*	KPC-2	CMY-113	-	TEM?	-	11	11	0	21	22	-1	21	20	1	21	23	-2	22	23	-1	11	10	1	20	22	-2	21	20	1	23	20	3	21	20	1
33	*E. coli*	KPC-2	-	-	TEM-1	-	21	22	-1	27	29	-2	27	26	1	28	29	-1	28	29	-1	25	24	1	29	28	1	30	29	1	29	31	-2	31	29	2
134	*E. coli*	KPC-2	-	-	CARB-23	-	25	25	0	28	30	-2	29	28	1	28	30	-2	30	28	2	18	20	-2	24	23	1	23	22	1	25	25	0	25	25	0
2	*K. pneumoniae*	KPC-2	-	CTX-M-15	SHV-110/OXA-9/OXA-1/TEM-1	+	15	15	0	21	9	12	21	12	9	21	8	13	21	11	10	16	16	0	21	6	15	20	10	10	20	6	14	20	11	9
7	*K. pneumoniae*	KPC-2	-	CTX-M-15	SHV-11/OXA-1	+	14	15	-1	23	6	17	22	11	11	23	6	17	21	10	11	15	14	1	25	6	19	24	8	16	25	6	19	24	9	15
12	*K. pneumoniae*	KPC-2	-	-	SHV-11/OXA-9/TEM-168	-	15	15	0	21	20	1	16	17	-1	19	20	-1	18	16	2	15	14	1	23	23	0	22	20	2	24	24	0	22	19	3
23	*K. pneumoniae*	KPC-2	-	-	SHV-11/OXA-9/TEM-163/TEM-185	-	6	6	0	19	12	7	17	6	11	18	14	4	19	6	13	7	8	-1	18	12	6	17	6	11	19	12	7	18	6	12
65	*K. pneumoniae*	KPC-2	-	-	SHV-11	-	15	15	0	24	25	-1	22	21	1	25	26	-1	22	19	3	14	18	-4	27	27	0	25	21	4	25	27	-2	24	23	1

69	*K. pneumoniae*	KPC-2	-	-	SHV-11	-	9	8	1	24	24	0	24	24	0	24	25	-1	26	24	2	9	7	2	27	26	1	27	25	2	28	28	0	28	26	2
70	*K. pneumoniae*	KPC-2	-	-	SHV-11	-	19	19	0	26	28	-2	25	25	0	26	28	-2	26	25	1	15	15	0	25	24	1	25	22	3	26	25	1	25	22	3
74	*K. pneumoniae*	KPC-2	-	CTX-M-15	SHV-11/OXA-1	+	17	16	1	25	10	15	26	12	14	25	6	19	25	10	15	13	15	-2	24	6	18	22	9	13	25	6	19	23	10	13
79	*K. pneumoniae*	KPC-2	-	-	SHV-11	-	16	16	0	25	26	-1	23	23	0	27	28	-1	26	26	0	15	17	-2	27	27	0	24	22	2	26	27	-1	25	23	2
81	*K. pneumoniae*	KPC-2	-	CTX-M-15	SHV-11/OXA-1	+	16	15	1	22	9	13	20	11	9	23	9	14	22	10	12	15	14	1	24	6	18	23	9	14	25	6	19	23	10	13
97	*K. pneumoniae*	KPC-2	CMY-16	CTX-M-15/SHV-66	OXA-9/OXA-10/TEM-1	+	6	6	0	16	6	10	16	6	10	16	6	10	16	6	10	6	6	0	17	6	11	17	6	11	17	6	11	17	6	11
99	*K. pneumoniae*	KPC-2	-	SHV-66	OXA-9/TEM-1	-	12	11	1	21	13	8	19	8	11	19	12	7	19	6	13	9	8	1	20	13	7	20	6	14	21	6	15	21	6	15
104	*K. pneumoniae*	KPC-2	-	CTX-M-15	SHV-11/OXA-1	+	16	16	0	23	6	17	21	10	11	22	6	16	23	9	14	16	16	0	23	6	17	21	11	10	24	6	18	20	6	14
135	*K. pneumoniae*	KPC-2	-	CTX-M-15	SHV-1/TEM-1	+	6	6	0	17	10	7	18	14	4	14	8	6	18	17	1	6	6	0	15	6	9	17	14	3	15	6	9	17	13	4
137	*K. pneumoniae*	KPC-2	-	CTX-M-15	SHV-1/TEM-1	+	6	6	0	17	9	8	18	16	2	18	9	9	20	15	5	6	6	0	16	6	10	21	14	7	17	6	11	20	14	6
153	*K. pneumoniae*	KPC-2	-	CTX-M-15	SHV-1/OXA-1	+	20	18	2	23	13	10	23	18	5	24	14	10	22	16	6	19	19	0	23	11	12	22	14	8	23	6	17	22	16	6
161	*K. pneumoniae*	KPC-2		CTX-M-15	SHV-1/TEM-192	+	6	6	0	19	8	11	19	15	4	16	9	7	21	16	5	6	6	0	16	6	10	21	15	6	17	7	10	22	15	7

162	*K. pneumoniae*	KPC-2	-	CTX-M-15	SHV-1/TEM-166/TEM-192	+	6	6	0	19	9	10	18	14	4	19	8	11	21	16	5	6	6	0	16	6	10	19	14	5	16	6	10	19	14	5
164	*K. pneumoniae*	KPC-2	-	CTX-M-15	SHV-1/TEM-166/TEM-192	+	6	6	0	18	10	8	19	15	4	18	8	10	19	15	4	6	6	0	15	6	9	19	15	4	17	6	11	20	15	5
170	*K. pneumoniae*	KPC-2	-	CTX-M-15	SHV-1/TEM-166/TEM-192	+	6	6	0	18	8	10	20	13	7	17	8	9	19	15	4	6	6	0	17	6	11	21	15	6	18	6	12	20	15	5
181	*K. pneumoniae*	KPC-2	-	CTX-M-15	SHV-1/TEM-166/TEM-192	+	6	6	0	18	6	12	19	16	3	17	8	9	21	17	4	6	6	0	17	6	11	20	15	5	17	6	11	21	15	6
189	*K. pneumoniae*	KPC-2	-	CTX-M-15	SHV-1/TEM-1	+	10	10	0	20	12	8	20	17	3	15	6	9	17	14	3	6	6	0	16	7	9	18	14	4	17	9	8	18	14	4
196	*K. pneumoniae*	KPC-2	-	CTX-M-15	SHV-1/TEM?	+	6	6	0	18	8	10	20	16	4	16	8	8	21	14	7	6	6	0	17	6	11	19	14	5	17	6	11	20	14	6
197	*K. pneumoniae*	KPC-2	-	CTX-M-15	SHV-1/TEM?	+	6	6	0	18	8	10	21	16	5	15	8	7	20	15	5	6	6	0	18	6	12	21	16	5	19	6	13	22	15	7
204	*K. pneumoniae*	KPC-2	-	CTX-M-55/SHV-66	-	+	6	6	0	10	6	4	10	6	4	9	6	3	11	6	5	6	6	0	15	6	9	17	6	11	14	6	8	17	6	11
87	*E. coli*	KPC-3	-	-	OXA-1/TEM-9	-	24	24	0	27	28	-1	24	23	1	26	28	-2	24	22	2	22	21	1	27	27	0	23	21	2	28	27	1	23	24	-1
209	*E. coli*	KPC-3		CTX-M-15/CTX-M-65	TEM-135/SHV-11	+	18	18	0	18	9	9	17	11	6	20	11	9	16	10	6	23	23	0	26	13	13	24	19	5	26	15	11	25	19	6
6	*K. pneumoniae*	KPC-3	-	-	SHV-?/TEM-1	-	18	18	0	21	22	-1	15	15	0	20	21	-1	17	15	2	19	19	0	22	22	0	16	6	10	22	22	0	17	15	2
11	*K. pneumoniae*	KPC-3	-	CTX-M-15	SHV-28/OXA-9/OXA-1/TEM-1	+	12	12	0	22	12	10	18	15	3	22	13	9	17	14	3	13	12	1	22	11	11	18	12	6	23	13	10	18	13	5

13	*K. pneumoniae*	KPC-3	-	-	SHV-11	-	16	15	1	22	21	1	16	15	1	22	23	-1	17	16	1	17	16	1	24	24	0	18	16	2	25	25	0	20	16	4
14	*K. pneumoniae*	KPC-3	-	-	SHV-11/OXA-9/TEM-1	-	12	10	2	20	20	0	17	17	0	20	20	0	17	17	0	7	6	1	18	19	-1	17	15	2	19	20	-1	18	16	2
30	*K. pneumoniae*	KPC-3	-	-	SHV-11	-	8	6	2	19	19	0	15	15	0	19	20	-1	16	15	1	6	6	0	18	19	-1	17	14	3	20	21	-1	18	17	1
42	*K. pneumoniae*	KPC-3	-	-	SHV-11/TEM-?	-	6	6	0	20	20	0	16	15	1	20	21	-1	16	16	0	6	6	0	19	20	-1	16	15	1	19	21	-2	16	16	0
62	*K. pneumoniae*	KPC-3	-	-	SHV-1/OXA-9/TEM-1	-	6	6	0	20	20	0	16	17	-1	18	19	-1	16	15	1	6	6	0	19	19	0	15	18	-3	19	19	0	16	16	0
75	*K. pneumoniae*	KPC-3	-	-	SHV-11/OXA-9/TEM-1	-	6	6	0	17	17	0	14	13	1	17	17	0	12	14	-2	6	6	0	17	18	-1	14	13	1	19	18	1	15	14	1
109	*K. pneumoniae*	KPC-3	-	CTX-M-15	SHV-28/OXA-1/OXA-9/TEM-1	+	22	19	3	28	13	15	25	14	11	31	14	17	29	13	16	19	20	-1	25	6	19	21	13	8	26	13	13	23	12	11
118	*K. pneumoniae*	KPC-3	-	-	OKP-B-6	-	20	20	0	24	25	-1	20	19	1	25	27	-2	21	20	1	22	21	1	24	24	0	20	20	0	24	27	-3	21	20	1
149	*K. pneumoniae*	KPC-3	-	CTX-M-15	SHV-28/OXA-1/TEM-1	+	22	22	0	22	14	8	20	18	2	23	15	8	19	14	5	20	22	-2	22	6	16	19	13	6	22	14	8	20	14	6
163	*K. pneumoniae*	KPC-3	-	-	SVH-11	-	10	8	2	19	20	-1	16	14	2	18	19	-1	13	14	-1	8	6	2	18	19	-1	16	15	1	18	20	-2	16	15	1
132	*K. pneumoniae*	KPC-50	-	-	SHV-85/TEM-1a	-	13	13	0	19	19	0	9	6	3	21	22	-1	11	6	5	13	11	2	18	18	0	10	6	4	21	20	1	13	6	7
201	*K. pneumoniae*	OXA-48/KPC-2	-	CTX-M-55	OXA-1/SHV-11	+	6	6	0	8	6	2	10	6	4	6	6	0	11	6	5	6	6	0	15	6	9	17	6	11	16	6	10	19	6	13

206	*K. pneumoniae*	OXA-48/KPC-3	-	-	SHV-1	-	6	6	0	6	6	0	6	6	0	6	6	0	6	6	0	6	6	0	18	18	0	15	15	0	18	18	0	14	15	-1
**MBL + KPC producers**																																				
203	*P. rettgeri*	KPC-2/VIM-19	CMY-4	PER-4	OXA-9	-	6	6	0	6	6	0	6	6	0	6	6	0	6	6	0	16	13	3	18	6	12	21	6	15	27	8	19	29	6	23
205	*K. pneumoniae*	KPC-3/NDM-1	-	-	SHV-11	-	6	6	0	6	6	0	6	6	0	6	6	0	6	6	0	9	9	0	18	19	-1	14	13	1	19	20	-1	15	14	1
208	*K. pneumoniae*	KPC-3/NDM-1	-	-	SHV-11	-	6	6	0	8	6	2	6	6	0	8	6	2	6	6	0	6	6	0	20	20	0	18	17	1	20	21	-1	20	18	2

N.p., not performed.
